# TNFR2 Agonism: Basic Science and Promising Treatment for Multiple Sclerosis and Related Diseases

**DOI:** 10.3390/ijms26167839

**Published:** 2025-08-14

**Authors:** Denise L. Faustman, Miriam Davis, Willem M. Kuhtreiber

**Affiliations:** 1 Laboratory of Immunobiology, Massachusetts General Hospital, and Harvard Medical School, Room 3602, MGH-East, Bldg 149, 13th Street, Boston, MA 02114, USA; wkuhtreiber@mgh.harvard.edu; 2 Laboratory of Immunobiology, Massachusetts General Hospital, MGH-East, Bldg 149, 13th Street, Boston, MA 02129, USA; miriamruthdavis@gmail.com

**Keywords:** TNFR2, multiple sclerosis, autoimmunity, T-regulatory cells, T-effector cells, CNS, Alzheimer’s disease, Parkinson’s disease

## Abstract

The three pathological hallmarks of multiple sclerosis (MS) are inflammation, demyelination, and progressive neurodegeneration. None of the approved disease-modifying therapies for MS counters all three pathologies, and, more specifically, none is approved for neuroprotection. Axonal loss is the most significant contributor to chronic and irreversible disability in MS. A tantalizing molecular target has emerged to uniquely counter all three MS pathologies: tumor necrosis factor receptor 2 (TNFR2). Agonism or activation of TNFR2 has been shown in MS models to induce immunosuppression, oligodendrocyte precursor differentiation, and neuroprotection. Further, in basic science studies stemming from the past 15 years, TNFR2 agonism is known to be a strong inducer of T-regulatory cells (Tregs). Treg cells, and especially those expressing TNFR2, are known to confer the strongest suppression per cell type. TNFR2 is even more attractive as a therapeutic target because of its restricted expression by only a handful of CNS and immune cell subsets, thereby minimizing the likelihood of systemic and other adverse effects. Recent antibody design work suggests many of the hurdles of Treg agonism may have been overcome. This review covers the current treatment landscape for MS, the basic science of TNFR2, the rationale for and evidence behind TNFR2 agonism to treat multiple sclerosis, the design of potent TNFR2 agonist antibodies, and the treatment applications for other neurological, autoimmune, or inflammatory diseases.

## 1. Introduction

The three pathological hallmarks of multiple sclerosis (MS) are inflammation, demyelination, and progressive neurodegeneration [1]. An entirely new molecular target has emerged to treat all three MS pathologies: tumor necrosis factor receptor 2 (TNFR2). Its activation has been shown in MS models to trigger immunosuppression, oligodendrocyte precursor differentiation, and neuroprotection [2,3,4,5]. What makes TNFR2 even more attractive as a therapeutic target is its restricted expression by only a handful of CNS and immune cell subsets, thereby minimizing the likelihood of systemic and other adverse effects [6,7].

This review covers the current treatment landscape for MS and its limitations, the basic science of TNFR2, and the rationale for TNFR2 agonism for treating multiple sclerosis and other neurological, autoimmune, or inflammatory diseases.

### Overview of Disease-Modifying Treatments for Multiple Sclerosis and Limitations

Significant advances have been made in developing disease-modifying therapies (DMTs) for MS. There are currently about 23 approved DMTs, most of which target neuroinflammation by T- and B-lymphocytes, the primary effectors of disease pathology [8,9]. DMTs are designed to interrupt the course of disease by reducing relapses and short-term disability. While many have succeeded in improving the quality of life, DMTs do not completely prevent disease progression or relapse [10]. Most DMTs work by such mechanisms as the sequestration of lymphocytes in lymph nodes, shifting CD4+ T cells from a T_H_1 (pro-inflammatory) to a T_H_2 (anti-inflammatory) cellular phenotype, induction of lymphocyte cell death by disruption of DNA synthesis, and inhibition of lymphocyte entry into the CNS by binding to its integrin subunit, among others [8,11].

The approved DMTs fall into three main categories based on differences in efficacy, routes of administration, and target tissue, among other factors [8]. Injectable therapies (interferon-B and glatiramer acetate) are considered first-line therapies with efficacies of around 29% to 34% vs. placebo in terms of relapse reduction. Oral therapies (three sphingosine 1-phosphate receptor modulators, fumarates, and teriflunomide) form the second tier with moderate to high efficacy of around 36–58% over 2 years. The highest efficacy therapies are monoclonal antibodies (natalizumab, ocrelizumab, alemtuzumab, and ublituximab), which have efficacies of around 68% versus a placebo or by 46% to 59% versus active comparators such as interferon beta-1a [8,12].

None of the approved or investigational DMTs work through the agonism of TNFR2. This emerging target may be unique in its potential to counter all three pathologies of MS, with minimal adverse effects by virtue of its limited bodily distribution on highly immunosuppressive T-regulatory cells (Tregs), oligodendrocyte precursor cells, microglia, astrocytes, neuronal stem cells, and injured neurons.

Axonal loss remains the source of chronic and irreversible disability in MS [10], considering that none of the DMTs has been explicitly approved for neuroprotection [13]. Some evidence of neuroprotection has been found, as a secondary outcome, for siponimod. This DMT shows less brain volume loss than the placebo (0.28% vs. 0.46% loss within 12 months, respectively) [14]. A member of the same class of drugs, fingolimod, may also be neuroprotective [15]. Evidence of neuroprotection is being sought for experimental drugs in the pipeline or already approved [16,17,18].

There are two different overall approaches to treating MS [19]. One is to start therapy with agents of lower treatment efficacy at disease onset followed by subsequent escalation to more effective agents as the disease progresses. The other approach is to initiate treatment with high-efficacy therapies. Several randomized Phase 3 studies comparing high and low efficacy drugs find that early treatment works best—in terms of up to 60% lower annualized relapse rates and reduced disability—with high-efficacy vs. low-efficacy medications [20]. The high-efficacy first-line approach is now recommended in treatment guidelines [21,22].

The safety of high-efficacy MS therapies has been studied in a network meta-analysis of 33 randomized clinical trials [23]. The evaluation focused on natalizumab, fingolimod, alemtuzumab, cladribine, ocrelizumab, ofatumumab, ozanimod, and, potentially, ponesimod. No differences between them were seen for serious adverse events, except for cladribine with higher side effects (17.3%) versus a comparison to ocrelizumab (10.3%). In addition, a comparison between ofatumumab and ocrelizumab found ofatumumab had over 16.6% higher side effects. Alemtuzumab displayed a higher rate of any type of adverse event (98.2%) versus all others in this group of high-efficacy therapies. No differences were apparent between this group of therapies for several individual adverse events that are common, such as upper respiratory infections, nasopharyngitis, fatigue, and nausea.

## 2. Basic Science of TNFR2

Tumor necrosis factor (TNF) is a multifunctional cytokine and master regulator of the immune system [24]. Excess TNF has been implicated in the pathophysiology of multiple sclerosis since the late 1980s. MS patients were shown to have elevated levels of TNF at the site of active lesions, and TNF levels correlated with MS lesion severity [25,26].

TNF works through two transmembrane receptors with opposing functions: TNFR1 (also known as p55 and TNFRSF1A) and TNFR2 (also known as p75 or TNFRSF1B). TNFR1 signaling promotes inflammation, tissue degradation, and apoptosis, while TNFR2 signaling promotes tissue homeostasis, survival, and regeneration [24]. Thus, TNF is said to control the life and death of cells, depending on which receptor is activated [27].

**Figure 1:**
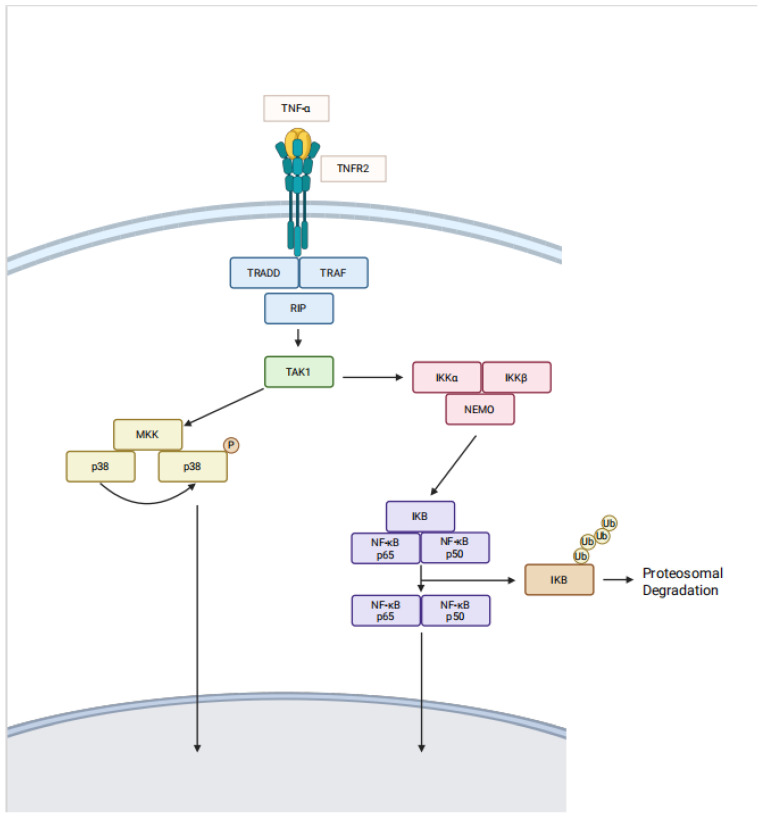
The signaling pathway for TNFR2 receptor signaling.

Since TNFR2+ expressing cells may also express TNFR1, receptor cross-talk [28], predominance, cell activation status, and cellular environment determine whether the cell assumes a pro-survival (TNFR2) vs. a pro-inflammatory phenotype/cell death (TNFR1) [27,29]. TNFR2 signaling relies on NF-kB, PI3K-Akt, and MAPK/ERK pathways [30,31,32] (Figure 1). TNFR1 uses an entirely different signaling pathway with agonism usually resulting in cell death due to the cytoplasmic death domain. The TNFR1 receptor also differs from the TNFR2 receptor in that TNFR1 is constitutively expressed on almost all cells in the human and is not associated with inducible expression. TNFR2, on the other hand, is expressed infrequently on most cell types and is an inducible receptor, meaning that at sites of inflammation its expression commonly elevates.

TNF is often mischaracterized as a pro-inflammatory cytokine largely because of the ubiquity of TNFR1 signaling, which is indeed pro-inflammatory; TNFR1 is constitutively expressed by nearly all cells of the body [24]. By contrast, TNFR2 has very restricted bodily distribution; it is only expressed by subsets of immune, CNS, and endothelial cells. More specifically, TNFR2 expression is largely restricted to highly immunosuppressive T-regulatory cells (Tregs) [33], macrophages [34], neural stem cells/progenitors in the brain’s subventricular zone and hippocampus [35,36], oligodendrocyte precursor cells [37], microglia, and astrocytes [38,39,40]. Most mature neurons express little to no TNFR2 under normal conditions, but neurons that are stressed or injured sometimes upregulate TNFR2, such as in neurodegenerative diseases and stroke [40].

While TNFR2 is only expressed by a handful of cell types, most if not all of them happen to play a role in multiple sclerosis: oligodendrocyte precursor cells and mature oligodendrocytes, microglia, macrophages, astrocytes, and Treg cells—each of which is described below.

Oligodendrocyte precursor cells (OPCs) are immature cells in the CNS that differentiate into mature oligodendrocytes, which form a myelin sheath by wrapping around axons. The sheath insulates axons for speedier impulse transmission. Mature oligodendrocytes generally express lower levels of TNFR2 than OPCs [41,42]. In MS, oligodendrocytes are targets of immune attack by T cells and B cells [1]. Stripped of myelin, axons can be spared by remyelination or they can be damaged or destroyed without remyelination. Remyelination in MS is generally carried out by OPCs [43].

Microglia are resident immune cells in the CNS that remove debris and pathogens. In MS, they can exert harmful effects (release of pro-inflammatory cytokines, reactive oxygen species, and nitric oxide that can damage myelin) and protective effects (clearance of myelin debris and release of growth factors to aid myelin repair) [44].

Macrophages are located throughout the body in virtually every tissue and organ. They can exist as resident macrophages in specific tissues or as circulating monocytes that differentiate into macrophages when they enter tissues. They are the foremost effectors of inflammation and demyelination in MS [44].

Astrocytes maintain the blood–brain barrier, regulate nutrients, and provide support to neurons. In MS, astrocytes are major participants in the formation and evolution of CNS lesions [45]. Astrocytes can become dysregulated in MS and can either exacerbate or help mitigate disease progression. Their harmful functions include astrogliosis and lesion formation, which can block remyelination and impair repair, as well as promote inflammation via cytokine release. Protective functions include tissue repair via release of growth factors that promote oligodendrocyte survival, proliferation, remyelination, and neuroprotection.

Treg cells (CD4+CD25+FOXP3+) are immunosuppressive. They are resident in the peripheral immune system, but in MS and other neurological diseases, they can infiltrate into the CNS to control inflammation by release of anti-inflammatory cytokines and by suppression of autoreactive T cells responsible for disease. They are more active in the peak phase of MS rather than at its origin [46]. Tregs may be dysfunctional in MS in at least two ways: their numbers may be depleted, or they can fail to effectively suppress autoreactive T cells [47]. Whether MS-related Treg dysfunctionality applies to the Treg subset that expresses TNFR2 remains unknown.

## 3. TNFR2 Boosting for Multiple Sclerosis

The rationale for TNFR2 agonism to treat multiple sclerosis stems from several lines of evidence, starting with the importance of TNF in MS pathogenesis. As early as 1988, TNF was found to damage oligodendrocytes and the myelin sheath in vitro [48]. Next, TNF was found to be increased in the blood and CSF of MS patients [49]. Studying post-mortem brains, MS patients were found to exhibit increased levels of TNF at the site of active lesions [25]. In 32 patients with chronic progressive MS studied prospectively over a 24-month period, TNF levels in CSF were shown to correlate with the severity of disease compared to patients with stable MS [26].

These early studies suggested that TNF blockade might be therapeutic in MS. However, three human studies or case reports suggested otherwise. First, administration of anti-TNF antibody infliximab, a nonselective inhibitor, transiently increased immune activation and increased disease severity in two MS patients [50]. Second, a large phase 2 placebo-controlled trial of the anti-TNF agent lenercept failed [51]. Lenercept is a recombinant soluble TNF receptor that binds to TNF, preventing it from interacting with its natural receptors (TNFR1 and TNFR2). Instead of curtailing exacerbations, the anti-TNF agent lenercept increased them in a nearly dose-dependent manner. Consequently, the trial was stopped prematurely. Third, a patient with juvenile rheumatoid arthritis developed a case of new-onset MS after being treated with etanercept, an anti-TNF antibody that signals through TNFR1 and TNFR2 [52]. Indeed, anti-TNF medications are now contraindicated in MS, based on this body of evidence. One possible explanation behind these findings is that non-selective anti-TNF drugs may not only have blocked the destructive effects of TNFR1 signaling but may also have blocked the protective effects of TNFR2 signaling [53].

The earliest studies to have assessed the role of TNFR2 in MS employed TNFR2 knockout mice. EAE (Experimental Autoimmune Encephalomyelitis) is the best studied animal model of MS. EAE mice with TNFR2 knockout exhibited a more severe course than wildtype mice and with an extreme degree of demyelination, suggesting a protective role for TNFR2 signaling in EAE [54]. TNFR1 signaling, on the other hand, was tied to symptom severity and demyelination. Arnett and colleagues [38] found that knockout mice lacking TNFR2 but not lacking TNFR1 failed to remyelinate in the cuprizone model of toxic demyelination and spontaneous remyelination (after cuprizone’s removal). Remyelination by this investigative team was assayed by the presence of the myelin basic protein and by electron microscopy. The team also found that TNFR2 but not TNFR1 is upregulated during the period of remyelination, although the results did not specify by which cell type. Relatedly, TNFR2 knockout mice develop EAE motor disease of greater severity than wildtype mice [55].

The first study to suggest that TNFR2 is involved in neuroprotection comes from an in vivo model of retinal ischemia in which TNFR2 knockout mice were shown to have aggravated neuronal cell death, assayed by counting retinal nuclei [56]. One of the first studies in EAE of TNFR2′s role in neuroprotection was by Brambilla and colleagues [37]. Using immunohistochemistry, they found TNFR2 localizes to spinal cord OPCs, injured oligodendrocytes, microglia/macrophages, and astrocytes but not neurons. In contrast, TNFR1 was found to localize to oligodendrocytes, astrocytes, and neurons and was found in association with axon damage. Remyelinating axons were observed with transmembrane TNF, which preferentially stimulates TNFR2, whereas soluble TNF, which preferentially stimulates TNFR1, is linked to axon damage and neurodegeneration. The study also examined two cases of postmortem human spinal cord with progressive MS, finding that TNFR2 was localized to activated microglia/macrophages and reactive astrocytes within white matter lesions, but it was not localized to neurons or mature oligodendrocytes. In a later study by the same team, EAE mice subject to conditional knockout with selective TNFR2 ablation were shown to increase myelin pathology and to increase axon loss by quantification of intact and degenerating axons [2]. The authors concluded that TNFR2 drives differentiation of OPCs, remyelination, and neuroprotection.

TNFR2 was also shown to suppress autoimmunity in EAE through the action of TNFR2+-expressing Tregs. Atretkhany and colleagues [3] created doubly humanized TNF/TNFR2 mice with the option for conditional TNFR2 inactivation in Treg cells. When TNFR2 was inactivated, the mice developed worse EAE, according to clinical scoring, and diminished capability to control Th17-mediated immune response, which plays a key role in autoimmunity and inflammation. The authors concluded that the beneficial effect of TNFR2 signaling in Tregs is to suppress CNS autoimmunity.

Using conditional knockouts, Gao and colleagues [57] discovered, in EAE mice, opposing functions of microglia vs. macrophage TNFR2. TNFR2 ablation in the microglia yielded earlier onset of EAE with heightened leukocyte infiltration, T cell activation, and demyelination in the CNS. TNFR2 ablation in macrophages, on the other hand, reduced CNS immune cell infiltration and suppressed EAE.

### 3.1. TNFR1 Antagonism Alone or Combined with TNFR2 Agonism in EAE Model

Several studies, all in EAE mice, found that TNFR1 antagonism can delay disease onset, attenuate disease activity, and preserve axons [37,58,59]. In TNFR1 knock-in mice, Fiedler and colleagues [5] evaluated the impact of TNFR1 antagonism (with human-specific H398) combined with TNFR2 agonism (with mouse-specific EHD2-sc-mTNF_R2_, which mimics membrane TNF). The result was reduced symptoms of EAE and less inflammatory infiltration, demyelination, and axonal degeneration. The two-pronged strategy prolonged neuron survival (in this case retinal ganglion cells), according to immunohistochemistry staining for neurofilaments, and fostered the phosphorylation of Akt and NF-kB, signaling related to neuroprotection. The authors also found that the two-pronged strategy worked better than each in isolation.

A sequential treatment approach with a TNFR2 agonist (EHD2-scTNF_R2_) followed by a TNFR1 antagonist (ATROSIMAB) was performed in TNFR-humanized EAE mice [60]. The investigators found that the sequential approach lessened paralysis symptoms and demyelination. The sequential approach was more effective than each approach alone. Interestingly, TNFR2 agonist alone yielded a fourfold increase in Treg infiltration in the spinal cord vs. saline control.

Unfortunately, the application to humans of an anti-TNFR1 strategy, whether alone or in combination with TNFR2 agonism, is unlikely to overcome safety concerns because of the widespread bodily distribution of TNFR1.

### 3.2. TNFR2 Agonism Alone in Culture or EAE Model

There are at least four published studies using TNFR2-specific agonists in culture or in the EAE model to improve the immune response in MS (Table 1).

The first study to examine the impact of TNFR2 agonism was conducted in OPCs cultured from the forebrain of transgenic mice expressing human TNFR2 [41]. The reason behind the use of transgenic mice was that the only available TNFR2 agonist at the time (TNC-scTNF_R2_) was specific for human TNFR2. The investigators first found that TNFR2 was expressed largely by OPCs, rather than by mature oligodendrocytes. Second, they showed that the agonist triggered OPC differentiation. Third, the agonist protected OPCs from hydrogen peroxide-induced oxidative stress. Finally, the agonist induced upregulation of anti-apoptotic genes.

A second study was conducted in EAE mice, given the TNFR2 agonist p53-sc-mTNF_R2_ [61]. The study used a non-pertussis toxin method to induce EAE because other studies revealed that pertussis toxin diminishes T-cell numbers and inhibits signaling through G protein-coupled receptors. The investigators found that male and female mice responded differently to TNFR2 agonism. The females showed a reduction in both sensory (mechanical hypersensitivity) and motor symptoms (using behavioral rating), as well as delay in motor symptom onset, whereas the males only showed reduction in sensory but not motor symptoms. Both sexes exhibited reduced cortical microglial activation indicative of a lower degree of chronic inflammation.

Ronin and colleagues [46] evaluated the impact of TNFR2 agonist STAR2 in EAE. The agonist, given from day 4 to day 18, led to a dramatic reduction in the EAE clinical score. Most of the study was devoted to determining the timing of Treg activity in EAE, finding that Tregs were more active at disease peak rather than at disease onset.

The most extensive study of the therapeutic potential of a TNFR2 agonist, EHD2-sc-mTNF_R2_, was conducted in EAE mice by Fischer and colleagues [4]. They found the following: TNFR2 agonism (vs. saline) prevented weight loss after motor symptom onset; it delayed the onset of motor deficits; reduced neuropathic pain, as shown by increased paw withdrawal threshold; expanded peripheral Treg numbers; reduced peripheral and central inflammation according to levels of pro-inflammatory cytokines and chemokines in blood and spinal cord; reduced T cell infiltration into the CNS, given that infiltration is a major driver of EAE pathology; and reduced spinal cord demyelination and neurodegeneration. The latter was assayed by a marker of axonal injury (i.e., accumulation of amyloid-beta precursor protein). Lastly, TNFR2 agonism induced proliferation of OPCs, and it increased differentiation of myelinating oligodendrocytes, suggesting that TNFR2 may cause remyelination.

### 3.3. How TNFR2 Agonism Might Protect Neurons in MS

TNFR2 agonism may mediate neuroprotection in MS, even though mature neurons normally express little to no TNFR2. There are several possible ways to protect neurons in MS—directly and indirectly. The direct method would be for MS neurons to upregulate TNFR2, which in turn would unleash pro-survival pathways, but this has not been studied in EAE/MS. The first of three indirect methods is by triggering TNFR2-expressing OPCs to differentiate into oligodendrocytes that remyelinate denuded axons. Remyelination saves those axons from degenerating, dying back, and causing the death of the entire neuron. The second indirect method is by tamping down on CNS inflammation via activation of TNFR2-expressing Tregs. Once activated, they can infiltrate into the CNS, where they can release anti-inflammatory cytokines and suppress autoreactive T cells [46]. The third indirect approach is by shifting TNFR2-expressing microglia and astrocytes from a pro-inflammatory to an anti-inflammatory phenotype. Their release of growth factors and anti-inflammatory cytokines or chemokines can protect neurons. These are among the approaches that will be discussed below in the context of TNFR2 agonism for other neurological diseases and autoimmune diseases.

### 3.4. TNFR2 Boosting for Other Neurological Diseases

Higher levels of soluble TNF (sTNFR2) are a common feature of neuroinflammatory and neurodegenerative conditions in addition to MS [62,63]. To counter the influence of TNFR1 signaling, which preferentially responds to soluble, as opposed to transmembrane, TNF, several studies have explored TNFR2 agonism as a therapeutic strategy in animal or in vitro models of Parkinson’s disease [64], neuropathic pain [65], traumatic injury [66], stroke [67] and Alzheimer’s disease [68,69]. We are unaware of any human trials for these indications.

One of the first studies of TNFR2 agonism for neuroprotection used the soluble human agonist TNC-scTNF_R2_ to rescue human neurons from oxidative stress-induced cell death [64]. Studying the human dopaminergic cell line LUHMES, the study first showed that TNFR2 agonism disrupted cell death pathways after exposure to hydrogen peroxide to simulate oxidative stress. Agonist action was dependent on signaling through the PI3K-PBK/Akt pathway. In addition, the same TNFR2 agonist was used to rescue neurons from induction of cell death by the 6-hydroxydopamine in vitro model of Parkinson’s disease. The authors concluded, “TNFR2-signaling by TNC-scTNF_R2_ appears a promising strategy to ameliorate neurodegenerative processes.”

In the next study by the same research team, Fischer and colleagues [65] showed that the same TNFR2 agonist (TNC-scTNF_R2_) led to Treg-mediated recovery from neuropathic pain. Studying mice spinal cord after chronic constriction injury, they first showed that genetic ablation of TNFR2 compromised neuronal regeneration and led to chronic nonresolving pain. The percentage of Treg cells was lower in injured mice in comparison to injured wildtype mice and uninjured TNFR2-ablated mice. No other immune cells were affected. When they introduced the TNFR2 agonist to injured mice not subject to genetic TNFR2 ablation, they first confirmed that Tregs and myeloid cells expressed the highest levels of TNFR2 in response to injury. Then, they showed that TNFR2 agonism improved two types of neuropathic pain: thermal hyperalgesia and tactile allodynia, according to behavioral assays. Agonism also yielded lower central and peripheral inflammation, with heightened numbers of Treg cells observed in the spinal cord. (There was no change in the number of microglia.) In a follow-up experiment they showed that recovery from pain was dependent on Tregs, because Treg depletion before injury was not associated with recovery from mechanical allodynia, and Treg depletion precluded therapeutic benefit from TNFR2 agonist.

The use of TNFR2 agonist (TNC-scTNF_R2_) was also evaluated in mice exposed to traumatic contusive injury to the spinal cord [66]. The team first showed that TNFR2 agonism protects neurons exposed in vitro to glutamate-induced excitotoxicity, a finding consistent with research showing neuronal TNFR2 signaling mediates neuroprotection from excitotoxicity in mice [70]. Then, they showed that agonist infused directly into the spinal cord of mice over a month-long period improved the locomotion of animals subject to contusive spinal cord injury. No changes were evident in the microglia studied by expression of glial fibrillary acidic protein or Iba-1, a biomarker of activated microglia. TNFR2 activation did not affect levels of TNF and other pro-inflammatory cytokines. Finally, TNFR2 agonism led to cortical neuron reorganization that contributed to recovery, based on sensory evoked potentials measured electrophysiologically.

In a mouse model of ischemic stroke, Thougaard and colleagues [67] studied the acute and chronic impact of the TNFR2 agonist NewSTAR2. Experimental stroke was induced by the permanent middle cerebral artery occlusion (pMACO) procedure. Animals were dosed with TNFR2 agonist or control antibody injected intraperitoneally starting at the time of pMACO. Two behavioral tests found that agonist-treated animals had better neuromuscular functional outcome soon after the procedure but not at later stages. No long-term change in gene expression of TNF, TNFR1, or TNFR2 was seen (although TNF expression showed transient upregulation). Systemic agonist activity affected circulating immune cell numbers (monocytes and neutrophils were increased, but CD45+CD11b^−^ cells were reduced) and transiently cut the number of microglia. Agonist treatment did not alter astrocyte and oligodendrocyte populations studied by flow cytometry. The authors concluded that “no significant long-term improvements” were seen after pMACO and NewSTAR2 treatment, in contrast to earlier studies described above in relation to MS and spinal cord injury. The differences were attributed to acute vs. chronic effects of injury.

Alzheimer’s disease, like other neurodegenerative diseases discussed here, is marked by elevated TNF levels, but TNF inhibition fails to treat the disease [71]. One research team used TNFR2 agonism to treat Alzheimer’s disease in two mouse models. In the first study [68], the investigators used the TNFR2 agonist NewStar2 in a transgenic Aβ-overexpressing mouse. Administration of the agonist centrally with an osmotic pump, as well as intraperitoneally, led to “drastic” reduction in amyloid β deposition and β-secretase I (BACE-1) expression levels. BACE-1 is the driver behind the production of Aβ peptides. The agonist also increased activation of microglia to increase phagocytotic uptake and degradation of Aβ plaques. The TNFR2 agonist delivered intraperitoneally exhibited a three-fold increase in the number of infiltrating Treg cells in the hippocampus compared to control mice. The agonist had a neuroprotective effect in cortical neurons in vitro. Finally, the agonist, after 6 weeks of treatment, was associated with behavioral improvement using several memory tests.

To make their findings more relevant to humans, the same research team [69] studied TNFR2 agonism (using NewStar2) in the same transgenic Aβ-overexpressing mouse, but this time with a fully human TNFR2 receptor, which is different from that in the mouse. The findings were remarkably consistent with those from the earlier experiment. The mice were rescued from memory-related cognitive impairments. The mice also showed a massive decrease in plaque load and in hippocampal beta-secretase 1 (BACE-1) expression levels relative to controls. Microglia were responsible for phagocytosis of Aβ deposits. The authors did not report on the Treg levels. One limitation of the study was that the mouse model lacks the presence of neurofibrillary tangles and thus does not completely mimic human pathology.

In contrast to the TNFR2 agonism studies, a new study [40] examined the consequences of inhibiting TNFR2 signaling—specifically by microglia—in response to ischemic stroke or spinal cord injury. Acute CNS injury stimulates rapid microglial activation and release of cytokines and growth factors to remove debris and other scavenger functions. But with prolonged activation, microglia upregulate TNFR2 to become the highest cellular expressors [72]. TNFR2 signaling by microglia is suspected to contribute to chronic CNS damage and worsening of functional outcome after stroke and spinal cord injury, according to earlier research by the investigators. Consequently, they sought to block microglial TNFR2 signaling by conditional knockout in mice. They found that TNFR2 ablation in microglia reduces ischemic lesion size, reduces levels of pro-inflammatory cytokines, and reduces leukocyte infiltration, but in a sexually dimorphic manner, with less microglial and macrophage activation seen in female than male mice. Ablation of TNFR2 also carried no functional impact according to four behavioral tests.

## 4. TNFR2 Boosting for Autoimmune and Inflammatory Diseases

Excessive TNF is a hallmark of many autoimmune and inflammatory diseases. Anti-TNF biologics have enjoyed widespread use for decades in chronic conditions like rheumatoid arthritis, inflammatory bowel diseases, psoriasis, and ankylosing spondylitis [63]. However, anti-TNF biologics are contraindicated or have limited utility for MS and type 1 diabetes, respectively, and can accelerate these diseases. In addition, although antibody drugs might be thought to bind and inactivate TNF, this may not be the case, and these new drug complexes instead might actually be more potent for the paradoxical induction of Tregs though only through the TNFR2 pathway. This means anti-TNF drugs in some cases might actually not work by soaking up TNF but instead inhibit or promote pro-inflammatory processes through stimulation of both TNFR1 and TNFR2 [63]. This lack of selectivity has left patients with a range of adverse effects, such as gastrointestinal upset, opportunistic infections, new onset autoimmunity, and even behavioral disturbance, among others [73]. Another issue is a significant lack of efficacy, with around 10–30% of patients not responding (primary non-responders) or developing a progressive loss of efficacy affecting 23–46% (secondary non-response), with the percentage varying by disease and a host of other factors [74,75].

These challenges have propelled the search for agonistic TNFR2-specific therapies for autoimmune or other inflammatory conditions. One approach is to use TNFR2 agonism to expand the pool of highly potent Treg cells, a rare Treg subset that accounts for 5–10% of CD4+ T cells and that plays a major regulatory role in suppressing the immune response. The allure of this approach is three-fold: it can expand the rare pool of highly potent Tregs to reach clinical significance; it can remove the underlying disease process; and it can promote repair and regeneration, as noted earlier for MS and neurological diseases. We have shown that TNFR2 acts as a master control switch for Treg cell expansion by an agonist or for Treg inhibition by an antagonist [76]. TNFR2 antagonism is highly promising for cancer, which features excess immunosuppression [76]. In fact, there are at least two TNFR2 antagonists to have reached Phase 2 clinical trials in the cancer field [63].

Clinical applications of Treg cells have been thwarted by standard Treg ex vivo expansion protocols, because they yield heterogeneous progeny, i.e., mixed populations of CD4+ T cells, which are risky for clinical trials and thus are likely to encounter regulatory barriers. Instead, we have shown that TNFR2 agonism expands Treg cells in a homogeneous manner to produce progeny that are as highly immunosuppressive as the original population. Our research has shown that TNFR2 agonism can expand Tregs into a homogeneous population which expresses the same 14 cell surface markers [76]. The study also showed that TNFR2 agonism suppresses a major Treg cell target: T-effector (or Teff) cells (a mixed population of both cytotoxic CD8+ T-cells and CD4+ T-helper cells).

Type 1 diabetes features a lifelong Treg cell defect; namely, by an increase in resting Tregs (rTregs, or CD4 + CD25 + Foxp3 + CD45RO) and a paucity of activated Tregs (aTregs, or CD4 + CD25 + Foxp3 + CD45RA) compared to nondiabetic controls. We have shown that TNFR2 agonism corrects this defect through an increase in aTregs capable of suppressing autoreactive CD4+T cells in a dose-dependent way [77]. The downstream signaling in culture on human cells maps the pathways to highly desirable Treg transcription factors for maximal potency [77]. An earlier study by our laboratory showed that TNFR2 agonism resulted in the selective destruction of autoreactive T cells specific for insulin, a pathogenic population contributing to Type 1 diabetes [78].

Graft-versus-host disease (GVHD) is a major cause of morbidity and mortality after organ, tissue, and cellular transplantation. Immune cells from the graft mount an excessive response to the recipient’s own tissues. A single injection of the TNFR2 agonist NewSTAR2 has been shown five days later to raise Treg numbers by more than 300% and has been shown to enhance their suppressive activity [79]. In this study, the TNFR2 agonist also protected mice from developing lethal acute GVHD. A similar TNFR2 agonist, STAR2, expanded Treg numbers and protected recipient mice from acute GVHD [80]. A related study showed that Treg-mediated control of GVHD was completely blocked by TNFR2 antagonism or by TNFR2-deficient Tregs [81].

Research in animal models has also found that TNFR2 agonism holds potential against colitis [82,83], psoriasis, psoriatic arthritis, and inflammatory skin disorders [84,85,86], and autoimmune arthritis [87,88,89,90]. In animal models, various forms of TNFR2 agonism have not been associated with any reported toxicity and, in our own primate studies, we see no systemic toxicity even after months of dose escalation studies suggesting this approach might confer an excellent safety profile.

## 5. Design of Optimal TNFR2 Antibodies for Inflammation

Tregs are a rare lymphocyte subtype that show promise for treating infectious disease, allergy, graft-versus-host disease, autoimmunity, and asthma. Clinical applications of Tregs have not been fully realized because standard methods of expansion ex vivo produce heterogeneous progeny consisting of mixed populations of CD4 T cells. Heterogeneous progenies are risky for human clinical trials and thus face significant regulatory hurdles. With the goal of producing homogeneous Tregs, we developed a novel expansion protocol targeting tumor necrosis factor receptors (TNFRs) on Tregs. In in vitro studies, a TNFR2 agonist was found superior to standard methods in proliferating human Tregs into a phenotypically homogeneous population defined by the presence of 14 cell surface markers [76]. The TNFR2 agonist-expanded Tregs also were functionally superior in suppressing a key Treg target cell, cytotoxic T-lymphocytes. Targeting the TNFR2 receptor during ex vivo expansion and possible in vivo expansion is a new means for producing homogeneous and potent human Tregs for clinical opportunities.

A trimeric ligand bound to three receptors forms the basic unit of signaling of TNF superfamily signaling including TNFR2 signaling. The structure of the archetypical member of the TNFSF, TNF, is a trimer. Each monomer forms a classic “jelly roll” structure composed of parallel sheets that define the TNF homology domain (THD). The individual monomers self-assemble into noncovalent homotrimers. The assembled trimers resemble a “cork” that is wider on the top and narrower on the bottom. Despite low to moderate sequence homology, all TNFSF members show high structural homology. The corresponding receptor monomers bind on the outside at the interface between two ligand receptor monomers. This is critically important for receptor activation, as it ensures that only an intact trimeric ligand can trigger signaling, because even the loss of a single ligand monomer results in the loss of two receptor-binding sites.

Receptor clustering is required for efficient signaling and for receptor pre-assembly on the cell surface. Receptor monomers interact through the so-called PLAD formed by the N-terminal and the CRD1 domains of the receptor (Pre-Ligand Assembly Domain). From the parallel dimer structure, a model of surface arrangement has been proposed showing parallel dimers arranged in a tightly packed hexagonal arrangement with room only for ligand binding. This model concurs with the need for PLAD interactions.

## 6. Conclusions and Future Directions

TNFR2 agonism holds significant promise for treating MS, as well as other CNS conditions, including autoimmune, inflammatory and/or neurodegenerative diseases. The approach stands out for its potential for safely addressing all three pathological hallmarks of MS, based on research in MS models. There are no marketed TNFR2 agonists, but some are in preclinical or early phase clinical trials for autoimmunity but not MS specifically.

## Figures and Tables

**Table 1:** Summary of Major Studies of TNFR2 Agonism in MS Models.

Study	TNFR2 Agonist	Model	Findings	Limitation(s)
Maier et al., 2013 [41]	TNC-scTNF_R2,_ which is specific for human TNFR2	OPCs cultured from forebrain of transgenic mice expressing human TNFR2	-Agonist triggered OPC differentiation -Agonist protected OPCs from hydrogen peroxide-induced oxidative stress -Agonist triggered upregulation of anti-apoptotic genes	Culture study
Pegoretti et al., 2023 [60]	p53-sc-mTNF_R2_	EAE	-Sex difference found in response to agonist, with females showing reduction in sensory & motor symptoms and delay in motor symptom onset, while males only showed reduction in sensory symptoms -Both sexes showed reduction in chronic cortical inflammation	Agonist is selective for mice
Ronin et al., 2021 [46]	Star2 (Original construct)	EAE	-Agonist reduced EAE clinical score -TNFR2-expressing Tregs found more active at peak EAE rather than disease onset	Agonist is selective for mice
Fischer et al., 2019 [4]	EHD2-sc-mTNF_R2_	EAE	-Agonist prevented weight loss after motor symptom onset -Agonist delayed onset of motor deficits -Agonist reduced neuropathic pain -Agonist expanded peripheral Treg numbers -Agonist reduced central and peripheral inflammation -Agonist reduced T cell infiltration into the CNS, which curtails EAE pathology -Agonist reduced spinal cord demyelination and neurodegeneration -Agonist triggered proliferation of OPCs and increased differentiation of oligodendrocytes	Agonist is selective for mice
